# Neuroendocrine Regulation of Plasma Cortisol Levels During Smoltification and Seawater Acclimation of Atlantic Salmon

**DOI:** 10.3389/fendo.2022.859817

**Published:** 2022-04-21

**Authors:** Brett M. Culbert, Amy M. Regish, Daniel J. Hall, Stephen D. McCormick, Nicholas J. Bernier

**Affiliations:** ^1^ Department of Integrative Biology, University of Guelph, Guelph, ON, Canada; ^2^ U.S. Geological Survey, Eastern Ecological Science Center, S.O. Conte Anadromous Fish Research Laboratory, Turners Falls, MA, United States; ^3^ Department of Biology, University of Massachusetts, Amherst, Amherst, MA, United States

**Keywords:** adrenocorticotropic hormone, arginine vasotocin, corticotropin-releasing factor, *Salmo salar*, urotensin 1, cortisol

## Abstract

Diadromous fishes undergo dramatic changes in osmoregulatory capacity in preparation for migration between freshwater and seawater. One of the primary hormones involved in coordinating these changes is the glucocorticoid hormone, cortisol. In Atlantic salmon (*Salmo salar*), cortisol levels increase during the spring smoltification period prior to seawater migration; however, the neuroendocrine factors responsible for regulating the hypothalamic-pituitary-interrenal (HPI) axis and plasma cortisol levels during smoltification remain unclear. Therefore, we evaluated seasonal changes in circulating levels of cortisol and its primary secretagogue—adrenocorticotropic hormone (ACTH)—as well as transcript abundance of the major regulators of HPI axis activity in the preoptic area, hypothalamus, and pituitary between migratory smolts and pre-migratory parr. Smolts exhibited higher plasma cortisol levels compared to parr across all timepoints but circulating ACTH levels were only elevated in May. Transcript abundance of preoptic area corticotropin-releasing factor b1 and arginine vasotocin were ~2-fold higher in smolts compared to parr in February through May. Smolts also had ~7-fold greater hypothalamic transcript abundance of urotensin 1 (*uts-1a*) compared to parr in May through July. When transferred to seawater during peak smolting in May smolts rapidly upregulated hypothalamic *uts-1a* transcript levels within 24 h, while parr only transiently upregulated *uts-1a* 96 h post-transfer. *In situ* hybridization revealed that *uts-1a* is highly abundant in the lateral tuberal nucleus (NLT) of the hypothalamus, consistent with a role in regulating the HPI axis. Overall, our results highlight the complex, multifactorial regulation of cortisol and provide novel insight into the neuroendocrine mechanisms controlling osmoregulation in teleosts.

## 1 Introduction

Diadromous fishes must migrate between freshwater and seawater as part of their natural life history (1, 2). This represents a considerable challenge as—among other changes—fish must shift from preventing the passive loss of ions in freshwater to actively excreting ions in saltwater (3). To accomplish this, diadromous fishes undergo several major physiological changes prior to undergoing these migrations (3, 4). While a number of hormones are involved in the coordination of these changes, both in preparation for and following transitions between freshwater and seawater (5–7), the glucocorticoid hormone cortisol has emerged as one of the major endocrine regulators of osmoregulatory processes during these periods. Many studies investigating the physiological roles of cortisol during diadromous migrations have focused on Atlantic salmon (*Salmo salar*) because cortisol levels increase as Atlantic salmon undergo smoltification—whereby fish transition from pre-migratory parr to migratory smolts—prior to seawater migration (8–10). This increase in cortisol levels elicits a number of osmoregulatory changes that help prepare smolts for the transition from freshwater to seawater (6, 11), such stimulating the activity of Na^+^-K^+^-ATPase (NKA) and other ion transporters in the gills (12–14). In contrast, slow growing parr that do not reach an upper size threshold by the end of winter do not undergo smoltification (15, 16) and their cortisol levels remain low throughout the spring (9, 17). Additionally, while smolts only moderately increase their cortisol levels when transferred to seawater (17), parr exhibit a large cortisol response that is consistent with their reduced capacity to osmoregulate and survive in seawater (7, 18). Yet, despite the well-established roles of cortisol during diadromous migrations, the neuroendocrine mechanisms regulating cortisol levels during these periods remain unclear.

In teleost fishes, cortisol synthesis is regulated by the hypothalamic-pituitary-interrenal (HPI) axis (19, 20). Activation of the HPI axis is primarily controlled by corticotropin-releasing factor (CRF) neurons located within the hypothalamic and preoptic regions of the brain (21, 22). These neurons project to corticotropes located in the rostral pars distalis of the pituitary (23, 24) where CRF stimulates production of pro-opiomelanocortin (POMC) *via* activation of CRF receptor 1 [CRF-R1; (22, 25)]. Once synthesized, POMC within corticotropes is converted to bioactive adrenocorticotropic hormone (ACTH) by prohormone convertase 1 [PC1; (26)] and subsequently released into circulation. While POMC is also produced by melanotropes in the pars intermedia of the pituitary, ACTH in the pars intermedia is quickly cleaved into α-melanocyte stimulating hormone and corticotropin-like intermediate peptide by PC2 (22, 26–29). Therefore, the pars intermedia does not contribute to circulating ACTH levels. When ACTH reaches the anterior kidney it binds to melanocortin 2 receptors (MC2R) located on the interrenal cells and stimulates the enzymatic conversion of cholesterol into cortisol (30, 31). Thus, activation of the HPI axis causes systemic cortisol levels to rise and ultimately increases the actions of cortisol throughout the body.

While CRF is widely regarded as the primary neuroendocrine regulator of HPI axis activity across vertebrates (21, 32), the teleost-specific genome duplication (33–35) resulted in two CRF paralogs in teleosts [CRF-a and CRF-b; (36–38)]. The sequence, tissue distribution, and attributed biological functions of CRF-b more closely resemble that of CRF in other vertebrates (21, 36, 37, 39); although, CRF-a also displays a high affinity for CRF-R1 (36) and may therefore contribute to the regulation of the HPI axis in teleosts. Additionally, another peptide in the CRF family, urotensin 1 (UTS-1), also has a high affinity for CRF-R1 (40–43) and populations of UTS-1 neurons located in the lateral tuberal nucleus (NLT) of the hypothalamus project to the pituitary in both goldfish [*Carassius auratus*; (44)] and white sucker [*Catostomus commersonii*; (45, 46)]. However, CRF-related peptides are not the only hormones involved in regulating HPI axis activity. Arginine vasotocin (AVT) is another hypophysiotropic hormone that is strongly implicated in the regulation of HPI axis activity in vertebrates (47–49), including teleosts (50–53). In most vertebrates (*e.g.*, mammals, birds, and reptiles), the effects of AVT (or arginine vasopressin (AVP) in mammals) on ACTH synthesis appear to be mediated by V1b-type receptors because they are the primary AVT/AVP receptor subtype expressed on corticotropes (54–57). However, teleosts do not appear to possess V1b-type receptors (58, 59). Instead, the effects of AVT on corticotropes in teleosts are likely regulated by either one or both of the two V1a-type paralogs found in teleosts [AVTR-V1a1 and -V1a2; (58, 59)] because the stimulatory effect of AVT on ACTH release during *in vitro* pituitary perfusions was most effectively blocked by a V1-type receptor antagonist in rainbow trout [*Oncorhynchus mykiss*; (50)]. Therefore, the activity of the HPI axis is likely regulated by several hypophysiotropic hormones but the relative contribution of each hormone remains unclear in teleosts.

Several studies have observed changes in HPI axis activity and cortisol production during seawater transitions, but there remains little consensus as to which neuroendocrine factor(s) are responsible for these changes. Previous work has shown that CRF-b appears to play an important role in acutely stimulating the HPI axis following seawater transitions (60–64), but its participation in the regulation of seasonal elevations in cortisol synthesis that occur prior to seawater migrations is less certain. McCormick et al. (8) found that preoptic area transcript abundance of CRF-b in Atlantic salmon decreased during the spring as fish underwent smoltification, despite observing elevated cortisol levels during this same period. Interestingly, Ebbesson et al. (65) detected an increase in CRF neuron proliferation in the preoptic area of smolting Atlantic salmon. However, Ebbesson et al. suggested that these changes were likely related to CRF contributing to the upregulation of thyroid hormone synthesis during smoltification (66, 67) because similar proliferative effects in CRF neurons were observed following treatment of non-anadromous, landlocked Atlantic salmon with L-thyroxine (65). Additionally, central injections of CRF increase circulating thyroxine levels in coho salmon smolts [*Oncorhynchus kisutch*; (68)], consistent with a role for CRF in regulating thyrotropes during smoltification. Therefore, it has been suggested that hypothalamic production of UTS-1 may assume a more prominent role in regulating HPI axis activity during major life history transitions—including smoltification (8) and spawning (69)—as well as acclimation to seawater (60). However, there is currently only limited support for the involvement of hypothalamic populations of UTS-1-expressing neurons in regulating the HPI axis.

To characterize the neuroendocrine mechanisms responsible for regulating cortisol levels during smoltification in Atlantic salmon, we evaluated seasonal changes in circulating cortisol and ACTH levels, as well as transcript abundance of the major neuroendocrine regulators of the HPI axis. We assessed changes in smolts and parr (the same age as smolts but too small to undergo smoltification) during the spring smoltification period and as they acclimated to seawater during peak smolting in May. We also assessed changes during the subsequent “desmoltification” that occurs over the summer when smolts remain in freshwater (70, 71). Specifically, we measured mRNA levels of *avt*, *crf-a1*, *crf-a2*, *crf-b1*, *crf-b2*, *uts-1a*, and *uts-1b* in the preoptic area and hypothalamus [*avt* was only measured in the preoptic area because the AVT neurons that project to the pituitary are almost exclusively found in the preoptic area in Atlantic salmon (72)]. We also assessed the relative abundance of *avtr-v1a2*, *crf-r1a*, *crf-r1b*, *pc1*, *pc2*, and *pomc-a1*, *-a2* and *-b* in the pituitary. We predicted that transcript abundance of the major neural regulators of HPI axis activity would be higher in smolts compared to parr during the spring because smolts have higher cortisol levels than parr. Additionally, we predicted that smolts would show dampened transcriptional responses of genes involved in HPI axis regulation during seawater acclimation because cortisol levels increase less in smolts than in parr following seawater transfer (17). Lastly, while transcriptional data have previously implicated hypothalamic UTS-1 in the regulation of HPI axis activity in salmonids (8, 60, 69), UTS-1 expression has not previously been localized to specific nuclei within the hypothalamus in any salmonid. Therefore, we performed *in situ* hybridization to localize UTS-1 expression in the diencephalon and establish whether it is expressed in neurons that project to the pituitary.

## 2 Materials and Methods

### 2.1 Experimental Animals and Housing

Juvenile Atlantic salmon were obtained from the Kensington State Hatchery (Kensington, CT, USA) and brought to the U.S. Geological Survey (USGS) S.O. Conte Anadromous Fish Research Laboratory (Turners Falls, MA, USA) in the fall of 2018. Fish were maintained in 1.7 m diameter tanks supplied with flow-through ambient Connecticut River water at a flow rate of 4 L min^−1^ and provided with continuous aeration. They were maintained under natural photoperiod conditions and fed to satiation (BioOregon, ME, USA) using automatic feeders. In December of 2018, fish were separated by size into parr [fork length (L_f_) ≤ 9.1 cm] and pre-smolt (L_f_ ≥10.5 cm) groups based on a previously established winter threshold for smolt development in this strain of Atlantic salmon (9). Each group was maintained in duplicate tanks containing ~100 fish and all groups experienced identical temperature regimes throughout the experiment. Fish were kept at ambient temperatures (2-4°C) through the winter and then kept at 8-9°C beginning on February 15. This temperature was maintained throughout the spring so that all sampling times would be at identical temperatures, to prevent effects of temperature on transcription. After May 30, fish experienced normal summer temperatures (maximum of 18.4°C) until being placed back on cooler water (9-10°C) on July 3. All fish rearing and sampling protocols were carried out in accordance with USGS institutional guidelines and protocol LSC-9096 that was approved by the USGS Eastern Ecological Science Center Institutional Animal Care and Use Committee.

### 2.2 Sampling

Twelve parr and twelve smolts were sampled on February 19^th^, April 1^st^, May 6^th^, and Ju1y 15^th^ 2019. To avoid potential tank effects, fish of each group (parr or smolt) were sampled from two separate tanks at each timepoint (*N* = 6 per tank). To evaluate the response of fish to seawater exposure, parr and smolts were placed into six 1-m diameter tanks (3 tanks of parr and 3 tanks of smolts) containing 28 ppt recirculating seawater during the week of May 6^th^, 2019. Tanks were held at 8.5-9.5°C and contained particle, biological, and charcoal filtration, and continuous aeration. Fish were fed daily to satiation, but food was withheld the day prior to each sampling. Twelve parr and twelve smolts were sampled after 24, 96, and 240 h of seawater exposure.

Food was withheld for 24 h prior to the sampling of fish, which occurred between 0900 and 1400 h Eastern Standard Time. All fish were killed *via* terminal anesthesia using NaHCO_3_ (12mM) buffered MS-222 (100 mg L^−1^; pH 7.0) after which fork length (L_F_) and mass were recorded. Blood was collected from the caudal vasculature using a 1 mL ammonium heparinized syringe, spun at 3,200 *g* for 5 min at 4°C, and plasma was collected. Four to six gill filaments were placed in 100 µl of ice-cold SEI buffer (150 mM sucrose, 10 mM EDTA, 50 mM imidazole, pH 7.3) for later measurement of Na^+^-K^+^-ATPase (NKA) activity. Brains were removed, and the hypothalamus, pituitary, and preoptic area were isolated as per Bernier et al. (73) for later RNA extraction. All samples were immediately flash frozen in dry ice prior to being stored at −80°C.

### 2.3 Physiological and Morphometric Parameters

NKA activity in gill homogenates was determined using a temperature-regulated microplate method as described by McCormick (74). Ten microliters of each sample were run in duplicate in 96-well microplates at 25°C and read at a wavelength of 340 nm for 10 min using a spectrophotometer (BioTek Synergy 2, BioTek). Values are expressed relative to protein concentration of the homogenate, which was determined using a BCA protein assay (Thermo Fisher Scientific). Plasma osmolality was measured using a vapor pressure osmometer (Vapro 5520, Wescor). Condition factor [*K*; (75)] was calculated as $K=100*\frac{mass}{L_{F}^{3}},$ where mass was in g and L_F_ was in cm.

### 2.4 Circulating Hormone Levels

Circulating levels of cortisol were measured using a validated direct competitive enzyme immunoassay (76), while ACTH levels were measured using a commercially available radioimmunoassay (MP Biomedicals) that had previously been validated for use with salmonid plasma (8, 60).

### 2.5 RNA Isolation and qPCR

Hypothalamus, preoptic area, and pituitary samples (8 parr and 8 smolt per timepoint) were homogenized in Ribozol reagent (VWR International) using a Precellys Evolution tissue homogenizer (Bertin Instruments). Following the manufacturer’s protocol, total RNA was extracted, and its quantity and purity was assessed using a NanoDrop 2000 spectrophotometer (Thermo Scientific). To remove genomic DNA, 1µg of RNA was treated with DNase (PerfeCTa DNase 1; QuantaBio) and reverse transcribed into complementary DNA (cDNA) using qScript cDNA SuperMix (QuantaBio). Gene-specific primers (**Table 1**) were used to perform quantitative polymerase chain reaction (qPCR) on a CFX96 system (BioRad) with SYBR green supermix (SsoAdvanced Universal; BioRad). All samples were run in duplicate and negative controls, including no template controls (where cDNA was replaced with water) and no reverse transcriptase controls (where RNA reverse transcriptase was replaced with water during cDNA synthesis) were included. Each reaction contained a total of 20 µl, which consisted of 10 µl of SYBR green, 5 µl of combined forward and reverse primers (0.2 µM [final]), and 5 µl of 10x diluted cDNA (200x dilution for *pomc-a1*, *a2*, and *b*). Cycling parameters included a 30 s activation step at 95°C, followed by 40 cycles consisting of a 3 s denaturation step at 95°C and a combined 30 s annealing and extension step at 60°C. Melt curve analysis was conducted at the end of each run to confirm the specificity of each reaction by the presence of a single peak. To account for differences in amplification efficiency, standard curves were constructed for each primer set using serial dilutions (4x) of pooled cDNA. To correct for differences in primer amplification efficiency, the average threshold cycle value for each fish was fit to the antilog of the respective gene-specific standard curve. To correct for minor variations in template input and transcriptional efficiency, we measured transcript abundance of 40S ribosomal protein S11 (*40s*) and elongation factor 1α (*ef1α*) as reference genes. Transcript abundance was normalized to *40s* in the preoptic area, *ef1α* in the hypothalamus, and the geometric mean of the two genes in the pituitary, as these approaches yielded the greatest stability between groups and across time within each tissue.

**Table 1:** Gene specific primers used for real-time PCR (qPCR) or *in situ* hybridization (ISH).

	Primer Sequence (5’ to 3’)	Amplicon Size (bp)	Efficiency (%)	Accession Number
**qPCR**				
*avt*	F: GAGCCGTTTGAAGGAGTTCAG	64	104	XM_014132971
	R: TTCAGTCTAAAGTGCTTCGTCC			
*avtr-v1a2*	F: TGATCTTCAGCGGGCATCTC	54	91	XM_014169886
	R: CAGCACGGGAAACAGAGAGT			
*crf-a1*	F: TCGCCGAACACATCTCCTG	74	101	XM_014139988
	R: TGCGTGAGCTGAAGTTGTAA			
*crf-a2*	F: GGTCAACAGGGCTTTACTTCA	69	103	XM_014190344
	R: AACCGATTGCTGTTACCGAC			
*crf-b1*	F: CTTGATCCATCACTCGTGGAAA	98	108	XM_014181363
	R: GTCAGGGGTTCAACGAGATC			
*crf-b2*	F: GAGGAAGGCAGCTCTCAACT	84	96	XM_014159556
	R: TCATGTCGGGATCAACAGGAA			
*crf-r1a*	F: AGCTACAGAGGCTGACAATGG	69	99	XM_014204418
	R: CTGTGCTGCCTTTGGCAAAG			
*crf-r1b*	F: CAGAGGGTCAGGATGACAATG	59	108	XM_014192620
	R: GTGGCAAAAAGTCAGCTCTTG			
*ef1a*	F: TCCTGCGGAGTCTCAAAACC	96	103	XM_014141923
	R: CGTTGGGTTCTTTTCCTGCG			
*pc1*	F: TCAATGACAACGACCCTGAC	69	106	XM_014132048
	R: ACCTGGTTCCATGCTTGTTC			
*pc2*	F: GTCAATGGTAGTCGCCGTGG	140	105	XM_014203849
	R: GTGTGTGTGGTCATAAAGGGC			
*pomc-a1*	F: TGGAAGGGGGAGAGGGAGAG	162	102	NM_001198575
	R: CGTCCCAGCTCTTCATGAAC			
*pomc-a2*	F: CTGGAGGCTGGGACTGCGGA	157	109	NM_001198576
	R: CGTCCCAGCTCTTCATGAAC			
*pomc-b*	F: GACTAAGGTAGTCCCCAGAACCCTCAC	103	108	NM_001128604
	R: GACAGCGGTTGGGCTACCCCAGCGG			
*uts-1a*	F: CAGTGTCTGTAGACCACGG	89	104	XM_014205273
	R: TATCACCAGCCTTCAGCAAC			
*uts-1b*	F: GCAGTCTACTACAATCGCCAT	103	101	XM_014144548
	R: AAACGGTGCCTTGATCGC			
*40s*	F: TTTCTGACTTCGGTCCGTCG	88	94	XM_014193076
	R: TAAGCCCTCTCGGTTTGTGC			
**ISH**				
*uts-1a*	F: GCAAGTGTCGTCAACAAGG	859	NA	XM_014205273
	R: GAAAAGGCTTCTTCTGCTGG			

avt, arginine vasotocin; avtr, arginine vasotocin receptor; crf, corticotropin-releasing factor; crf-r, corticotropin-releasing factor receptor; ef1a, elongation factor 1α; pc, prohormone convertase; pomc, pro-opiomelanocortin; uts, urotensin; 40s, 40S ribosomal protein S11; NA, not applicable.

A pilot study determined that expression of *avtr-v1a1* in the pituitary was generally low (amplified at ~34-35 cycles) and was ~4x less abundant than *avtr-v1a2*. Because of these differences in abundance, we focused on *avtr-v1a2* and did not measure transcript abundance of *avtr-v1a1* in the pituitary. This difference in pituitary expression of the *avtr-v1a* paralogs is consistent with the distribution patterns reported in rock hind [*Epinephelus adscensionis*; (77, 78)], but different than in Amargosa pupfish [*Cyprinodon nevadensis amargosae*; (58)] and the catfish *Heteropneustes fossilis* (79, 80).

### 2.6 *In Situ* Hybridization

We focused the *in situ* hybridization analysis on *uts-1a* because *uts-1a* was ~8x more abundant than *uts-1b* (in both the preoptic area and hypothalamus) and *uts-1a* showed greater transcriptional changes compared to *uts-1b* during qPCR analyses (both seasonally and in response to seawater transfer).

Using cDNA originating from the hypothalamus, a fragment of *uts-1a* was amplified using PCR (**Table 1**). Primer sets had T7 recognition sites (5’-*TAATACGACTCACTATAGGG*-3’) attached to the 5’ end of either the forward (sense probe) or reverse (anti-sense probe) primer. Each PCR product was isolated using a PureLink quick PCR purification kit (Invitrogen) and gel electrophoresis was used to confirm the presence of a single product of the correct size. The purified products were then used as template to synthesize digoxigenin-labeled (DIG-labeled) riboprobes using T7 RNA polymerase as per the manufacturer’s instructions (Roche).

Whole brains from smolts (*N* = 3) were collected in May and fixed overnight at 4°C in a solution of 4% paraformaldehyde (PFA) in phosphate-buffered saline (PBS; 137 mM NaCl, 2.7 mM KCl, 8 mM Na_2_HPO_4_, and 2 mM KH_2_PO_4_; pH 7.4). Fixed brains were then sequentially transferred over 3 d to 30% sucrose in PBS, 1:1 30% sucrose in PBS : Cryomatrix (Shandon; Thermo Scientific), and Cryomatrix at 4°C. Following cryoprotection, brains were embedded in fresh Cryomatrix and stored at -80°C until they were cut into 7 µm thick sections using a cryostat (Leica Biosystems). Sections were collected on Superfrost Plus glass slides (Fisher Scientific) and dried overnight at 37°C. The following day, sections were treated (all washes were carried out at room temperature unless otherwise indicated) with: 4% PFA in PBS (15 min with gentle shaking), PBS + 0.1% Tween 20 (PBT; 2 × 5 min), 6% H_2_O_2_ in PBT (5 min), PBT (3 × 5 min), proteinase K (1 µg mL^-1^) in PBT (15 min with gentle shaking), glycine (2 mg mL^-1^) in PBT (10 min with gentle shaking), PBT (2 × 5 min), 4% PFA/0.2% glutaraldehyde in PBT (15 min), and PBT (3 × 5 min). Sections were then prehybridized for 1 h at 65°C in hybridization buffer without riboprobe (50% formamide, 25% saline-sodium citrate buffer (SSC; 300 mM Na_3_C_6_H_5_O_7_, 3 M NaCl; pH 4.5), 1% sodium dodecyl sulfate (SDS), 500 µg mL^-1^ heparin, 50 µg mL^-1^ torula yeast RNA), followed by overnight incubation at 65°C with hybridization buffer containing DIG-labeled riboprobe (500 ng mL^-1^). After hybridization, sections were washed in 50% formamide, 20% SSC, 1% SDS for 3 × 15 min at 70°C, 50% formamide, 10% SSC for 3 × 15 min at 65°C, Tris-buffered saline (TBS; 150 mM NaCl, 2.5 mM KCl, 25 mM Tris-HCl; pH 7.5) + 0.1% Tween 20 (TBST) for 3 × 10 min, and TBST with 5% lamb serum for 1 h with gentle shaking. After blocking, slides were incubated overnight at 4°C with anti-DIG antibody conjugated to alkaline phosphatase (Roche; diluted 1:2000 in TBST with 1% lamb serum). The next morning, slides were washed with TBST (4 × 15 min) and NTMT (100 mM NaCl, 100 mM Tris-HCl, 50mM MgCl_2_, 0.1% Tween 20; pH 9.5) for 3 × 15 min and were then developed in NTMT (with 10% polyvinyl alcohol) containing 5-bromo-4-chloro-3-indolyl phosphate (BCIP; 0.188 mg/mL) and nitro blue tetrazolium (NBT; 0.375 mg/mL) in the dark for 16 h. Following development, sections were treated with NTMT for 2 × 15 min, PBT (pH 5.5) for 2 × 10 min, 4% PFA/0.1% glutaraldehyde in PBS for 15 min and PBS for 3 × 5 min. Sections were then dehydrated in a series of ethanol washes with increasing concentration, cleared using xylene, and coverslipped with mounting media (Permount; Fisher Scientific). A parallel set of sections was collected from each fish and processed using Nissl staining to confirm the plane of sectioning and position of cell nuclei. Briefly, sections were stained for 10 min in 0.1% cresyl violet containing 0.3% acetic acid, dehydrated in a series of ethanol washes with increasing concentrations, and mounted as described above. Images of all sections were acquired using differential interference contrast microscopy on a Nikon Eclipse 90i microscope with NIS-Elements AR 3.2 software. We followed the atlases of Peter et al. (81) and Meek and Nieuwenhuys (82) for identification of neuroanatomical structures and brain nuclei.

### 2.7 Statistical Analysis

Statistical analyses were performed using R [version 3.6.3; (83)]. All data are presented as means ± 1 standard error of the mean (SEM) and a significance level (α) of 0.05 was used for all tests. Outliers were excluded based on a 2x interquartile range threshold. When data did not meet the assumptions of normality and/or equal variance, data were transformed, or if the data could not be transformed to meet the assumptions, then analyses were performed using ranked data. Data were analyzed using two-way ANOVAs that included group (parr and smolt) and either month (February, April, May, and July) or time following seawater exposure (0h, 24h, 96h, and 240h), as well as the interaction between these factors. When significant differences were detected, Tukey’s HSD *post hoc* analysis was performed.

## 3 Results

### 3.1 Seasonal Patterns

#### 3.1.1 Physiology and Morphometry

**Figure 1:**
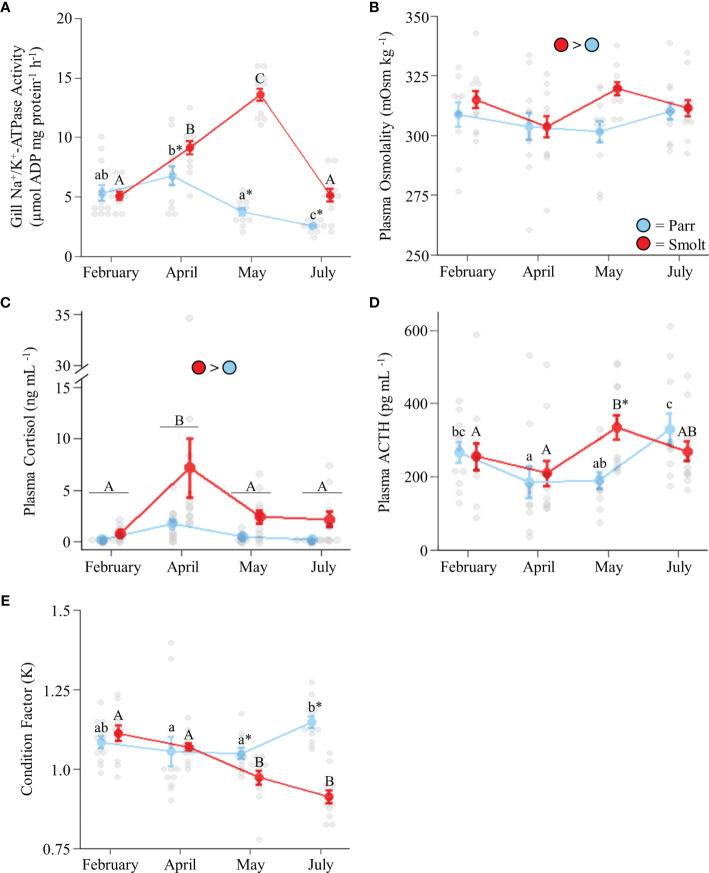
Seasonal changes in gill Na^+^/K^+^-ATPase activity **(A)**; plasma osmolality **(B)**, cortisol levels **(C)**, and adrenocorticotropic hormone levels (ACTH; **D**); and condition factor **(E)** in Atlantic salmon (*Salmo salar*). Parr are depicted in blue and smolts in red. Significant differences (p < 0.05) are depicted using either letters (across time; uppercase = within smolts, lowercase = within parr, underlined uppercase = overall time effect), filled oversized circles (between groups across all timepoints) or asterisks (between groups within a timepoint). Values are represented as means ± SEM and individual data points are shown. Parr and smolts were sampled on the same day for each timepoint, but data are offset for presentation purposes.

Branchial NKA activity in freshwater smolts increased ~2-fold through the spring and then returned to baseline levels in July, whereas NKA activity declined slightly through the spring in parr (**Figure 1A**; Group: *F*_1,85_ = 83.50, *p* < 0.001; Time: *F*_3,85_ = 31.29, *p* = 0.001; Group x Time: *F*_3,85_ = 19.30, *p* < 0.001). Plasma osmolality was slightly higher in smolts than parr throughout the spring and summer (**Figure 1B**; Group: *F*_1,85_ = 4.33, *p* = 0.04; Time: *F*_3,85_ = 1.59, *p* = 0.20; Group x Time: *F*_3,85_ = 1.87, *p* = 0.14), with the greatest difference (~6% higher in smolts) being observed in May. Smolts also had higher plasma cortisol levels than parr throughout the spring and summer (**Figure 1C**; Group: *F*_1,82_ = 43.25, *p* < 0.001; Time: *F*_3,82_ = 14.76, *p* < 0.001; Group x Time: *F*_3,82_ = 1.13, *p* = 0.34), especially in April when cortisol values of smolts were on average 4x those of parr. Plasma ACTH levels (**Figure 1D**; Group: *F*_1,84_ = 2.29, *p* = 0.13; Time: *F*_3,84_ = 5.44, *p* = 0.002; Group x Time: *F*_3,84_ = 3.34, *p* = 0.02) peaked in May for smolts, with levels that were ~1.5x higher than smolts in April and ~1.75x higher than parr in May. Plasma ACTH levels in parr increased to levels consistent with those of smolts by July. Lastly, condition factor (**Figure 1E**; Group: *F*_1,88_ = 16.78, *p* < 0.001; Time: *F*_3,88_ = 5.58, *p* = 0.001; Group x Time: *F*_3,88_ = 13.91, *p* < 0.001) in smolts declined to levels lower than parr in May and remained low through July, while condition factor in parr increased from May to July.

#### 3.1.2 mRNA Abundance

##### 3.1.2.1 Preoptic Area

**Figure 2:**
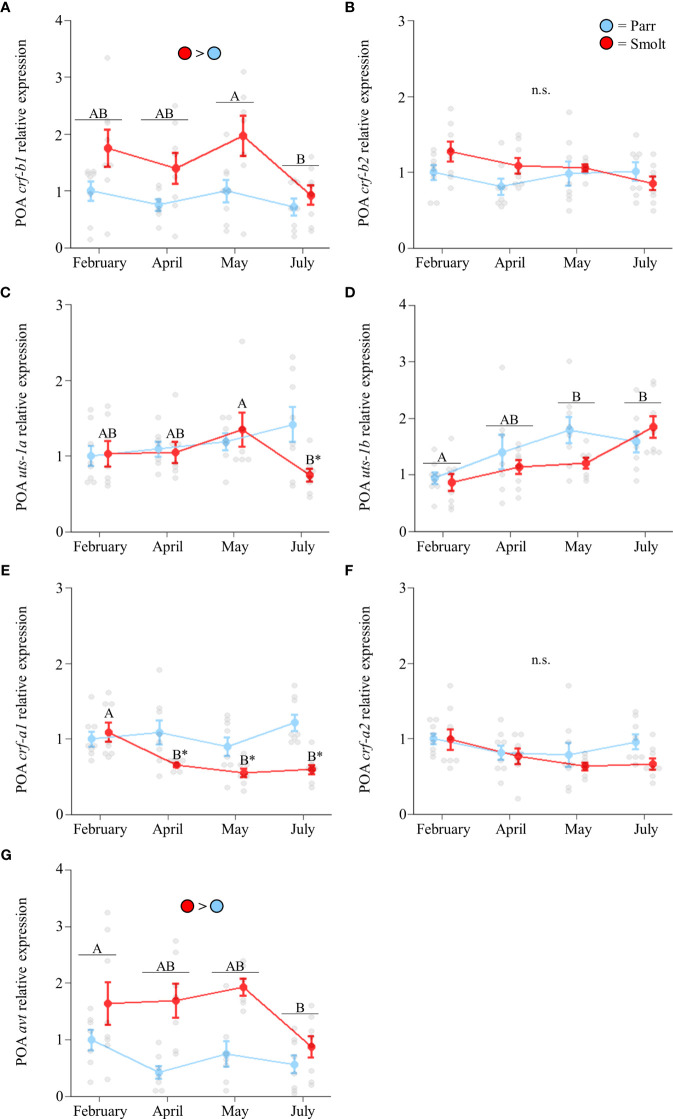
Seasonal changes in transcript abundance of corticotropin-releasing factor b1 (*crf-b1*; **A**) and b2 (*crf-b2*; **B**); urotensin 1a (*uts-1a*; **C**) and 1b (*uts-1b*; **D**); corticotropin-releasing factor a1 (*crf-a1*; **E**) and a2 (*crf-a2*; **F**); and arginine vasotocin (*avt*; **G**) in the preoptic area (POA) of Atlantic salmon (*Salmo salar*). Parr are depicted in blue and smolts in red. Significant differences (p < 0.05) are depicted using either letters (across time; uppercase = within smolts, underlined uppercase = overall time effect), filled oversized circles (between groups across all timepoints) or asterisks (between groups within a timepoint). Data are expressed relative to parr in February. Values are represented as means ± SEM and individual data points are shown. Parr and smolts were sampled on the same day for each timepoint, but data are offset for presentation purposes. n.s., no significant differences detected.

Transcript abundance of preoptic *crf-b1* (**Figure 2A**; Group: *F*_1,54_ = 15.33, *p* < 0.001; Time: *F*_3,54_ = 3.30, *p* = 0.03; Group x Time: *F*_3,54_ = 0.97, *p* = 0.41) was approximately twice as high in smolts compared to parr through the spring, with levels peaking in May and declining in July. Conversely, no changes in transcript abundance of *crf-b2* were detected (**Figure 2B**; Group: *F*_1,54_ = 2.08, *p* = 0.16; Time: *F*_3,54_ = 1.33, *p* = 0.28; Group x Time: *F*_3,54_ = 1.63, *p* = 0.19). Preoptic transcript abundance of *uts-1a* in smolts decreased in July (**Figure 2C**; Group: *F*_1,51_ = 1.70, *p* = 0.20; Time: *F*_3,51_ = 0.98, *p* = 0.41; Group x Time: *F*_3,51_ = 2.85, *p* = 0.047), while transcript abundance of *uts-1b* approximately doubled across the spring and summer in both parr and smolts (**Figure 2D**; Group: *F*_1,54_ = 1.49, *p* = 0.23; Time: *F*_3,54_ = 7.47, *p* < 0.001; Group x Time: *F*_3,54_ = 1.82, *p* = 0.15). Transcript levels of *crf-a1* in smolts decreased by 50% in April (**Figure 2E**; Group: *F*_1,55_ = 22.31, *p* < 0.001; Time: *F*_3,55_ = 4.36, *p* = 0.008; Group x Time: *F*_3,55_ = 4.14, *p* = 0.01), and these levels remained ~2-fold lower than parr through July. No significant changes in *crf-a2* were detected (**Figure 2F**; Group: *F*_1,55_ = 2.90, *p* = 0.09; Time: *F*_3,55_ = 2.60, *p* = 0.06; Group x Time: *F*_3,55_ = 0.74, *p* = 0.53). Transcript levels of *avt* (**Figure 2G**; Group: *F*_1,50_ = 24.43, *p* < 0.001; Time: *F*_3,50_ = 3.19, *p* = 0.03; Group x Time: *F*_3,50_ = 1.90, *p* = 0.14) were approximately twice as high in smolts compared to parr, but expression declined overall in July; especially in smolts.

##### 3.1.2.2 Hypothalamus

**Figure 3:**
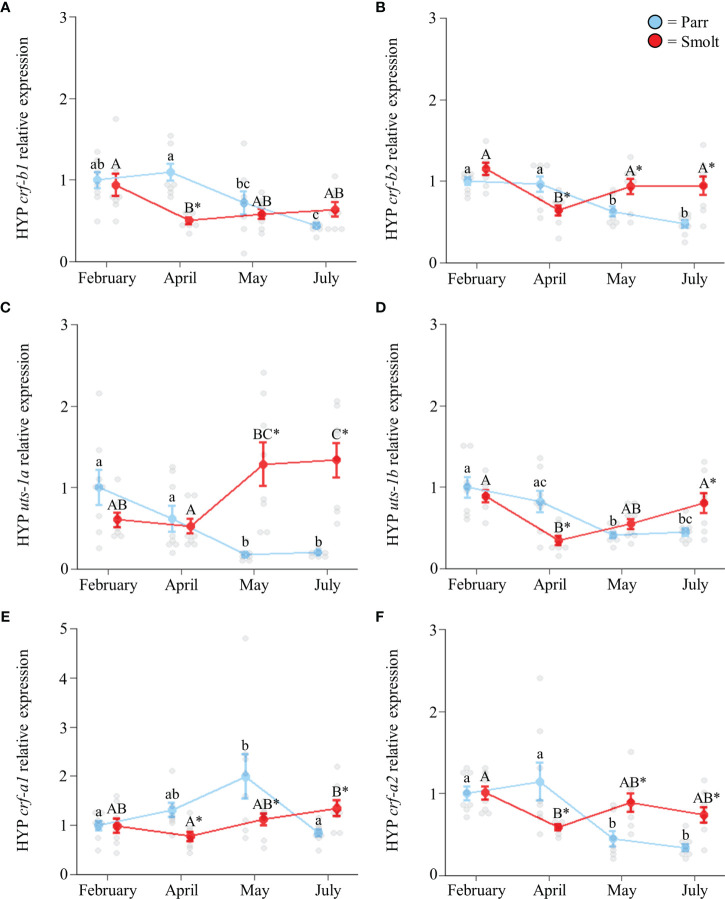
Seasonal changes in transcript abundance of corticotropin-releasing factor b1 (*crf-b1*; **A**) and b2 (*crf-b2*; **B**); urotensin 1a (*uts-1a*; **C**) and 1b (*uts-1b*; **D**); and corticotropin-releasing factor a1 (*crf-a1*; **E**) and a2 (*crf-a2*; **F**) in the hypothalamus (HYP) of Atlantic salmon (*Salmo salar*). Parr are depicted in blue and smolts in red. Significant differences (p < 0.05) are depicted using either letters (across time; uppercase = within smolts, lowercase = within parr) or asterisks (between groups within a timepoint). Data are expressed relative to parr in February. Values are represented as means ± SEM and individual data points are shown. Parr and smolts were sampled on the same day for each timepoint, but data are offset for presentation purposes.

Hypothalamic transcript abundance of *crf-b1* (**Figure 3A**; Group: *F*_1,54_ = 4.41, *p* = 0.04; Time: *F*_3,54_ = 7.06, *p* < 0.001; Group x Time: *F*_3,54_ = 5.45, *p* = 0.002) and *crf-b2* (**Figure 3B**; Group: *F*_1,55_ = 7.10, *p* = 0.01; Time: *F*_3,55_ = 8.16, *p* < 0.001; Group x Time: *F*_3,55_ = 9.41, *p* < 0.001) generally decreased across the spring and summer, especially in parr where levels in July were ~50% less than levels in February. Conversely, transcript abundance of *uts-1a* (**Figure 3C**; Group: *F*_1,55_ = 34.69, *p* < 0.001; Time: *F*_3,55_ = 2.04, *p* = 0.12; Group x Time: *F*_3,55_ = 18.84, *p* < 0.001) showed a clear divergence between parr and smolts, with ~6-fold higher values observed in smolts in May and July. Like *crf-b1* and *crf-b2*, seasonal changes in transcript abundance of *uts-1b* (**Figure 3D**; Group: *F*_1,54_ = 0.16, *p* = 0.69; Time: *F*_3,54_ = 9.64, *p* < 0.001; Group x Time: *F*_3,54_ = 9.38, *p* < 0.001), *crf-a1* (**Figure 3E**; Group: *F*_1,56_ = 2.47, *p* = 0.12; Time: *F*_3,56_ = 3.02, *p* = 0.04; Group x Time: *F*_3,56_ = 5.47, *p* = 0.002), and *crf-a2* (**Figure 3F**; Group: *F*_1,54_ = 6.99, *p* = 0.01; Time: *F*_3,54_ = 11.85, *p* < 0.001; Group x Time: *F*_3,54_ = 11.28, *p* < 0.001) were generally idiosyncratic, but tended to decrease across the spring and summer in both parr and smolts.

##### 3.1.2.3 Pituitary

**Figure 4:**
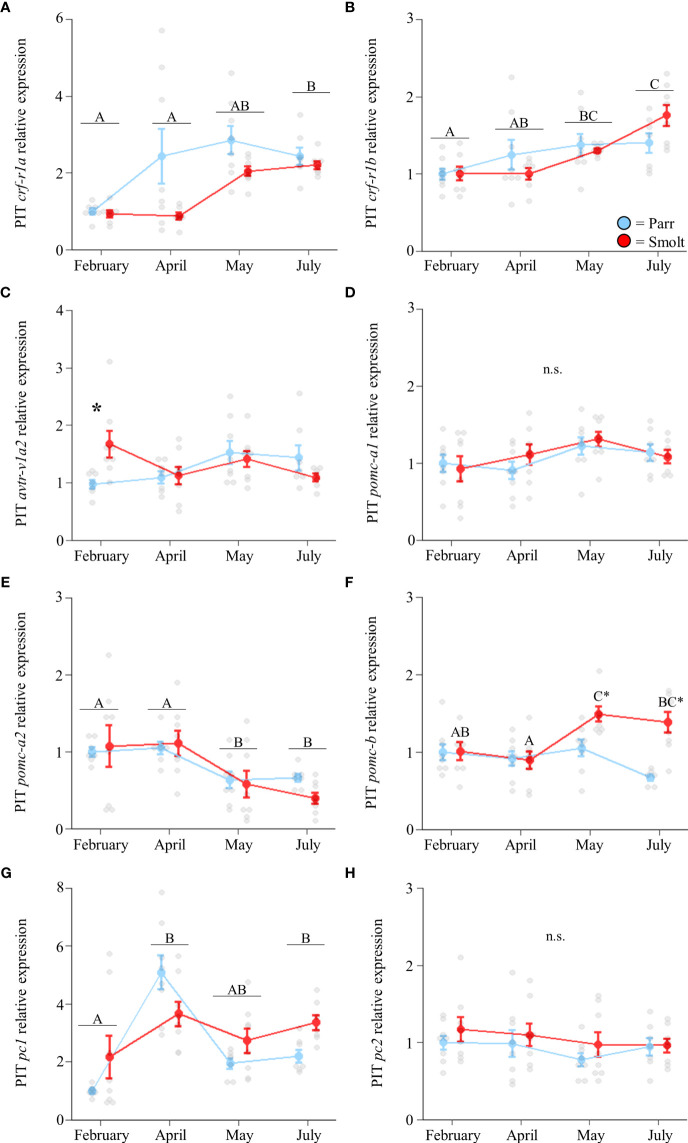
Seasonal changes in transcript abundance of corticotropin-releasing factor receptor 1a (*crf-r1a*; **A**) and 1b (*crf-r1b*; **B**); arginine vasotocin receptor v1a2 (*avtr-v1a2*; **C**); proopiomelanocortin a1 (*pomc-a1*; **D**), a2 (*pomc-a2*; **E**), and b (*pomc-b*; **F**); and prohormone convertase 1 (*pc1*; **G**) and 2 (*pc2*; **H**) in the pituitary (PIT) of Atlantic salmon (*Salmo salar*). Parr are depicted in blue and smolts in red. Significant differences (p < 0.05) are depicted using either letters (across time; uppercase = within smolts, underlined uppercase = overall time effect) or asterisks (between groups within a timepoint). Data are expressed relative to parr in February. Values are represented as means ± SEM and individual data points are shown. Parr and smolts were sampled on the same day for each timepoint, but data are offset for presentation purposes. n.s, no significant differences detected.

Transcript abundance of *crf-r1a* (**Figure 4A**; Group: *F*_1,51_ = 8.56, *p* = 0.005; Time: *F*_3,51_ = 13.07, *p* < 0.001; Group x Time: *F*_3,51_ = 2.11, *p* = 0.11) and *crf-r1b* in the pituitary (**Figure 4B**; Group: *F*_1,53_ = 0.02, *p* = 0.90; Time: *F*_3,53_ = 9.10, *p* < 0.001; Group x Time: *F*_3,53_ = 2.13, *p* = 0.11) increased through the spring in both parr and smolts, with *crf-r1a* displaying a larger increase than *crf-r1b*. Expression of *avtr-v1a2* was ~70% higher in smolts compared to parr in February, but there were no differences at any other timepoint (**Figure 4C**; Group: *F*_1,52_ = 0.46, *p* = 0.50; Time: *F*_3,52_ = 2.21, *p* = 0.10; Group x Time: *F*_3,52_ = 3.59, *p* = 0.02). No significant differences were detected in abundance of *pomc-a1* (**Figure 4D**; Group: *F*_1,54_ = 0.26, *p* = 0.61; Time: *F*_3,54_ = 2.72, *p* = 0.053; Group x Time: *F*_3,54_ = 0.60, *p* = 0.62), while transcript levels of *pomc-a2* decreased over time (**Figure 4E**; Group: *F*_1,53_ = 0.18, *p* = 0.67; Time: *F*_3,53_ = 7.67, *p* < 0.001; Group x Time: *F*_3,53_ = 0.55, *p* = 0.65). In contrast, transcript abundance of *pomc-b* only varied in smolts (**Figure 4F**; Group: *F*_1,53_ = 13.41, *p* < 0.001; Time: *F*_3,53_ = 4.34, *p* = 0.008; Group x Time: *F*_3,53_ = 5.29, *p* = 0.003) and displayed a ~50% increase in May which persisted into July. Transcript abundance of *pc1* peaked in April and remained elevated through May and July (**Figure 4G**; Group: *F*_1,51_ = 0.05, *p* = 0.83; Time: *F*_3,51_ = 3.87, *p* = 0.01; Group x Time: *F*_3,51_ = 0.23, *p* = 0.88), but did not differ between parr and smolt. No differences in *pc2* were detected (**Figure 4H**; Group: *F*_1,55_ = 1.80, *p* = 0.19; Time: *F*_3,55_ = 1.24, *p* = 0.30; Group x Time: *F*_3,55_ = 0.10, *p* = 0.96).

### 3.2 Following Seawater Exposure

#### 3.2.1 Physiology

**Figure 5:**
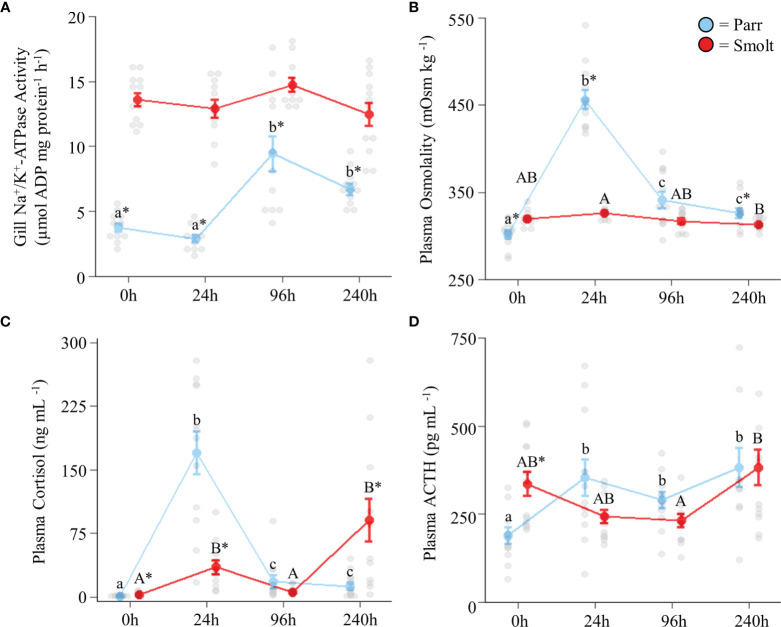
Effects of seawater exposure on gill Na^+^/K^+^-ATPase activity **(A)**; and plasma osmolality **(B)**, cortisol levels **(C)**, and adrenocorticotropic hormone levels (ACTH; **D**) in Atlantic salmon (*Salmo salar*). Parr are depicted in blue and smolts in red. Significant differences (p < 0.05) are depicted using either letters (across time; uppercase = within smolts, lowercase = within parr) or asterisks (between groups within a timepoint). Time 0h values are in freshwater, all others after exposure to 28 ppt seawater. Values are represented as means ± SEM and individual data points are shown. Parr and smolts were sampled on the same day for each timepoint, but data are offset for presentation purposes.

Parr doubled their branchial NKA activity after 96 h in seawater, whereas NKA activity in smolts remained stable and was higher than in parr across the entire 240 h period (**Figure 5A**; Group: *F*_1,84_ = 269.76, *p* < 0.001; Time: *F*_3,84_ = 20.93, *p* < 0.001; Group x Time: *F*_3,84_ = 16.43, *p* < 0.001). Parr also exhibited a ~50% increase in plasma osmolality after 24 h of seawater exposure, while smolts only displayed minor adjustments in their plasma osmolality across time (**Figure 5B**; Group: *F*_1,88_ = 3.78, *p* = 0.06; Time: *F*_3,88_ = 17.05, *p* < 0.001; Group x Time: *F*_3,88_ = 9.67, *p* < 0.001). While smolts initially had higher plasma osmolality values than parr in freshwater, parr generally had higher plasma osmolality values than smolts following transfer to seawater. Smolts exhibited a dampened cortisol response compared to parr (~6 vs. 300-fold increase) at 24 h post seawater transfer (**Figure 5C**; Group: *F*_1,84_ = 2.10, *p* = 0.15; Time: *F*_3,84_ = 51.88, *p* < 0.001; Group x Time: *F*_3,84_ = 12.92, *p <*0.001). However, cortisol levels in smolts increased ~14-fold compared to baseline values at the end of the 240 h seawater exposure trial. Plasma ACTH values in parr increased by ~75% after 24 h and plateaued, whereas circulating ACTH levels in smolts decreased by ~30% during the first few days following seawater transfer and then increased to levels consistent with parr by 240 h post-transfer (**Figure 5D**; Group: *F*_1,83_ = 0.08, *p* = 0.78; Time: *F*_3,83_ = 4.01, *p* = 0.01; Group x Time: *F*_3,83_ = 5.79, *p* = 0.001).

#### 3.2.2 mRNA Abundance

##### 3.2.2.1 Preoptic Area

**Figure 6:**
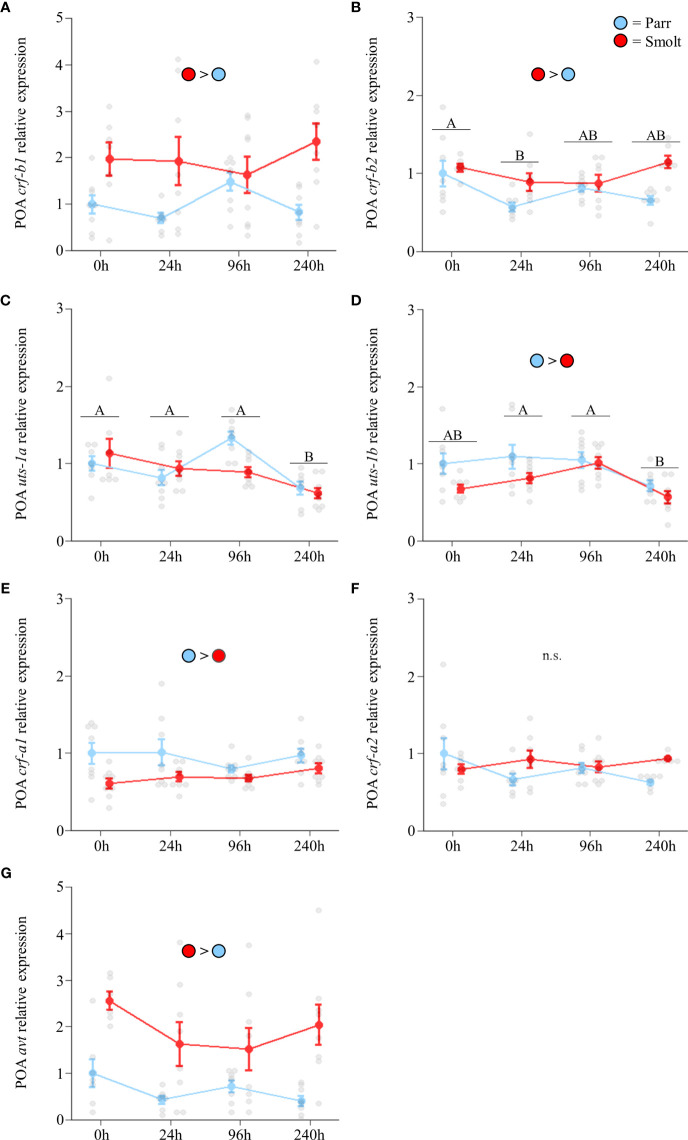
Effects of seawater exposure on transcript abundance of corticotropin-releasing factor b1 (*crf-b1*; **A**) and b2 (*crf-b2*; **B**); urotensin 1a (*uts-1a*; **C**) and 1b (*uts-1b*; **D**); corticotropin-releasing factor a1 (*crf-a1*; **E**) and a2 (*crf-a2*; **F**); and arginine vasotocin (*avt*; **G**) in the preoptic area (POA) of Atlantic salmon (*Salmo salar*). Parr are depicted in blue and smolts in red. Significant differences (p < 0.05) are depicted using either letters (across time; underlined uppercase = overall time effect), filled oversized circles (between groups across all timepoints). Time 0h values are in freshwater, all others after exposure to 28 ppt seawater. Data are expressed relative to parr at 0h. Values are represented as means ± SEM and individual data points are shown. Parr and smolts were sampled on the same day for each timepoint, but data are offset for presentation purposes. n.s., no significant differences detected.

Transcript abundance of preoptic *crf-b1* (**Figure 6A**; Group: *F*_1,54_ = 10.98, *p* = 0.002; Time: *F*_3,54_ = 0.51, *p* = 0.68; Group x Time: *F*_3,54_ = 1.96, *p* = 0.13) did not change following seawater exposure, with smolts maintaining levels approximately twice those of parr. Smolts also exhibited slightly higher transcript abundance of *crf-b2* (**Figure 6B**; Group: *F*_1,51_ = 13.47, *p* < 0.001; Time: *F*_3,51_ = 3.85, *p* = 0.01; Group x Time: *F*_3,51_ = 2.64, *p* = 0.06), with only minor fluctuations occurring over the course of the 240 h seawater exposure. Transcript abundance of *uts-1a* (**Figure 6C**; Group: *F*_1,53_ = 0.79, *p* = 0.38; Time: *F*_3,53_ = 10.11, *p* < 0.001; Group x Time: *F*_3,53_ = 2.67, *p* = 0.06) and *uts-1b* (**Figure 6D**; Group: *F*_1,55_ = 7.57, *p* = 0.008; Time: *F*_3,55_ = 7.38, *p* < 0.001; Group x Time: *F*_3,55_ = 0.80, *p* = 0.50) both decreased as fish acclimated to seawater, and transcript abundance of *uts-1b* was slightly higher overall in parr versus smolts. Transcript abundance of *crf-a1* was also slightly higher in parr over the duration of the seawater exposure (**Figure 6E**; Group: *F*_1,56_ = 13.04, *p* < 0.001; Time: *F*_3,56_ = 1.09, *p* = 0.36; Group x Time: *F*_3,56_ = 0.80, *p* = 0.50), while no differences were detected for *crf-a2* (**Figure 6F**; Group: *F*_1,51_ = 1.40, *p* = 0.24; Time: *F*_3,51_ = 0.63, *p* = 0.60; Group x Time: *F*_3,51_ = 2.56, *p* = 0.07). Abundance of *avt* (**Figure 6G**; Group: *F*_1,52_ = 21.40, *p* < 0.001; Time: *F*_3,52_ = 2.06, *p* = 0.12; Group x Time: *F*_3,52_ = 1.49, *p* = 0.23) was not significantly affected by seawater transfer and remained ~2x higher in smolts versus parr across all timepoints.

##### 3.2.2.2 Hypothalamus

**Figure 7:**
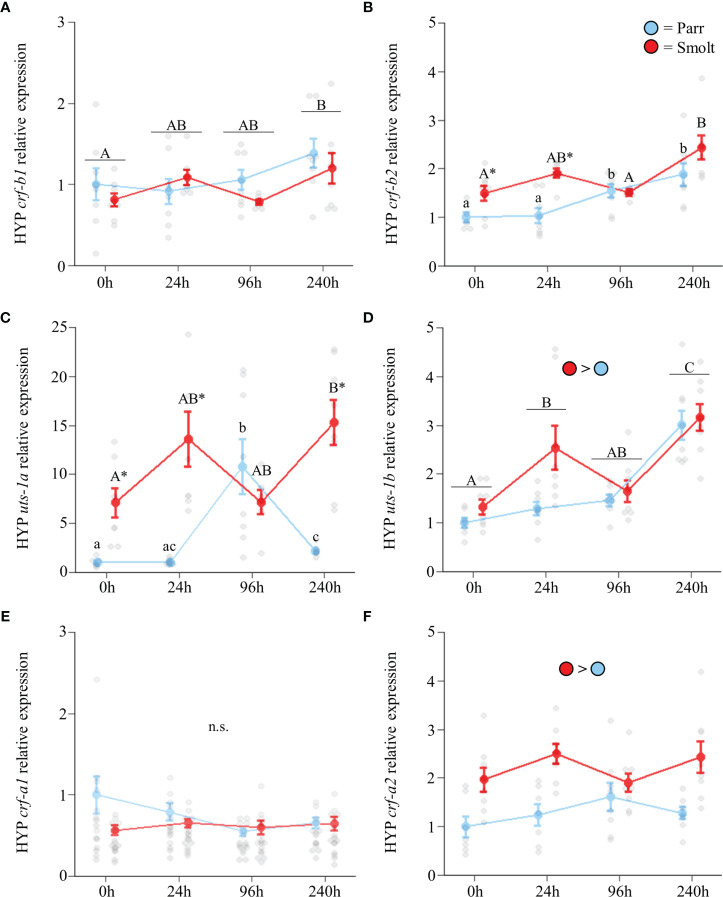
Effects of seawater exposure on transcript abundance of corticotropin-releasing factor b1 (*crf-b1*; **A**) and b2 (*crf-b2*; **B**); urotensin 1a (*uts-1a*; **C**) and 1b (*uts-1b*; **D**); and corticotropin-releasing factor a1 (*crf-a1*; **E**) and a2 (*crf-a2*; **F**) in the hypothalamus (HYP) of Atlantic salmon (*Salmo salar*). Parr are depicted in blue and smolts in red. Significant differences (p < 0.05) are depicted using either letters (across time; uppercase = within smolts, lowercase = within parr, underlined uppercase = overall time effect), filled oversized circles (between groups overall) or asterisks (between groups within a timepoint). Time 0h values are in freshwater, all others after exposure to 28 ppt seawater. Data are expressed relative to parr at 0h. Values are represented as means ± SEM and individual data points are shown. Parr and smolts were sampled on the same day for each timepoint, but data are offset for presentation purposes. n.s., no significant differences detected.

Hypothalamic transcript abundance of both *crf-b1* (**Figure 7A**; Group: *F*_1,53_ = 1.26, *p* = 0.27; Time: *F*_3,53_ = 3.00, *p* = 0.04; Group x Time: *F*_3,53_ = 0.87, *p* = 0.46) and *crf-b2* (**Figure 7B**; Group: *F*_1,53_ = 21.34, *p* < 0.001; Time: *F*_3,53_ = 11.23, *p* < 0.001; Group x Time: *F*_3,53_ = 3.56, *p* = 0.02) increased during seawater acclimation. Similarly, smolts and parr both upregulated hypothalamic *uts-1a* transcript abundance following seawater exposure (**Figure 7C**; Group: *F*_1,52_ = 108.67, *p* < 0.001; Time: *F*_3,52_ = 12.36, *p* < 0.001; Group x Time: *F*_3,52_ = 14.50, *p* < 0.001). However, while smolts doubled transcript levels of *uts-1a* within the first 24 h, parr exhibited a transient ~11-fold upregulation at 96 h post transfer. After 240 h of seawater exposure, both groups of fish maintained approximately twice the transcript levels of *uts-1a* compared to initial freshwater levels, with transcript levels being 7x greater in smolts relative to parr. Transcript abundance of *uts-1b* (**Figure 7D**; Group: *F*_1,54_ = 10.21, *p* = 0.002; Time: *F*_3,54_ = 26.00, *p* < 0.001; Group x Time: *F*_3,54_ = 2.56, *p* = 0.06) was higher in smolts and increased ~2-fold as seawater acclimation progressed. No differences in transcript abundance of *crf-a1* were detected (**Figure 7E**; Group: *F*_1,54_ = 2.49, *p* = 0.12; Time: *F*_3,54_ = 1.17, *p* = 0.33; Group x Time: *F*_3,54_ = 1.21, *p* = 0.31), while transcript levels of *crf-a2* were higher in smolts at all timepoints (**Figure 7F**; Group: *F*_1,53_ = 28.81, *p* < 0.001; Time: *F*_3,53_ = 1.17, *p* = 0.33; Group x Time: *F*_3,53_ = 1.71, *p* = 0.18).

##### 3.2.2.3 Pituitary

**Figure 8:**
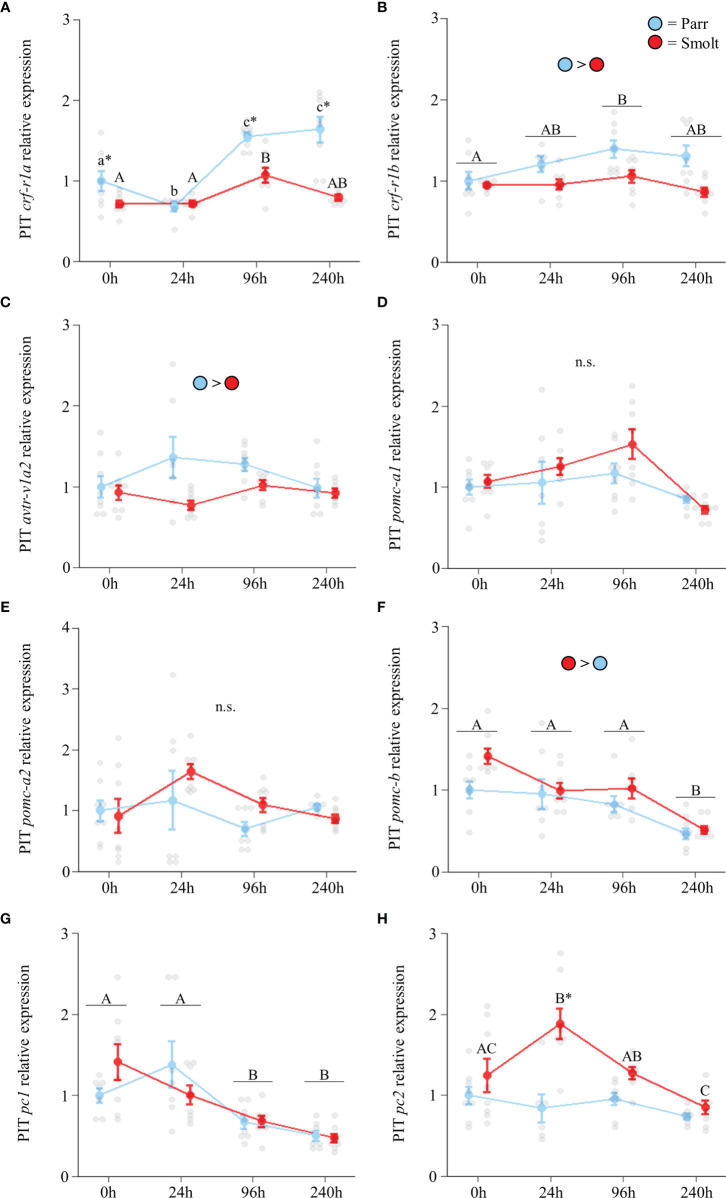
Effects of seawater exposure on transcript abundance of corticotropin-releasing factor receptor 1a (*crf-r1a*; **A**) and 1b (*crf-r1b*; **B**); arginine vasotocin receptor v1a2 (*avtr-v1a2*; **C**); proopiomelanocortin a1 (*pomc-a1*; **D**), a2 (*pomc-a2*; **E**), and b (*pomc-b*; **F**); and prohormone convertase 1 (*pc1*; **G**) and 2 (*pc2*; **H**) in the pituitary (PIT) of Atlantic salmon (*Salmo salar*). Parr are depicted in blue and smolts in red. Significant differences (p < 0.05) are depicted using either letters (across time; uppercase = within smolts, lowercase = within parr, underlined uppercase = overall time effect), filled oversized circles (between groups overall) or asterisks (between groups within a timepoint). Time 0h values are in freshwater, all others after exposure to 28 ppt seawater. Data are expressed relative to parr at 0h. Values are represented as means ± SEM and individual data points are shown. Parr and smolts were sampled on the same day for each timepoint, but data are offset for presentation purposes. n.s., no significant differences detected.

Transcript abundance of *crf-r1a* (**Figure 8A**; Group: *F*_1,53_ = 34.25, *p* < 0.001; Time: *F*_3,53_ = 20.51, *p* < 0.001; Group x Time: *F*_3,53_ = 6.52, *p* < 0.001) and *crf*-*r1b* (**Figure 8B**; Group: *F*_1,53_ = 116.37, *p* < 0.001; Time: *F*_3,53_ = 2.79, *p* = 0.049; Group x Time: *F*_3,53_ = 1.95, *p* = 0.13) increased during seawater exposure, with parr showing greater upregulation than smolts. Parr also had slightly higher abundance of *avtr-v1a2* (**Figure 8C**; Group: *F*_1,53_ = 6.57, *p* = 0.01; Time: *F*_3,53_ = 1.57, *p* = 0.21; Group x Time: *F*_3,53_ = 1.91, *p* = 0.14) across the entire seawater exposure period. No differences were detected in either *pomc-a1* (**Figure 8D**; Group: *F*_1,53_ = 0.03, *p* = 0.86; Time: *F*_3,53_ = 2.23, *p* = 0.09; Group x Time: *F*_3,53_ = 0.11, *p* = 0.95) or *pomc*-*a2* (**Figure 8E**; Group: *F*_1,54_ = 0.25, *p* = 0.61; Time: *F*_3,54_ = 0.74, *p* = 0.53; Group x Time: *F*_3,54_ = 1.65, *p* = 0.18); however, transcript abundance of *pomc-b* (**Figure 8F**; Group: *F*_1,53_ = 6.23, *p* = 0.02; Time: *F*_3,53_ = 21.61, *p* < 0.001; Group x Time: *F*_3,53_ = 0.50, *p* = 0.68) was higher in smolts and declined over time. Transcript abundance of *pc1* (**Figure 8G**; Group: *F*_1,52_ = 0.01, *p* = 0.94; Time: *F*_3,52_ = 20.31, *p* < 0.001; Group x Time: *F*_3,52_ = 1.33, *p* = 0.28) also declined as fish acclimated to seawater. Smolts responded to seawater exposure by transiently increasing transcription of *pc2* at 24 h (**Figure 8H** Group: *F*_1,53_ = 18.21, *p <*0.001; Time: *F*_3,53_ = 5.64, *p* = 0.002; Group x Time: *F*_3,53_ = 4.26, *p* = 0.009), but transcript abundance of *pc2* did not change in parr.

### 3.3 Localization of *uts-1a* Transcripts in the Diencephalon

**Figure 9:**
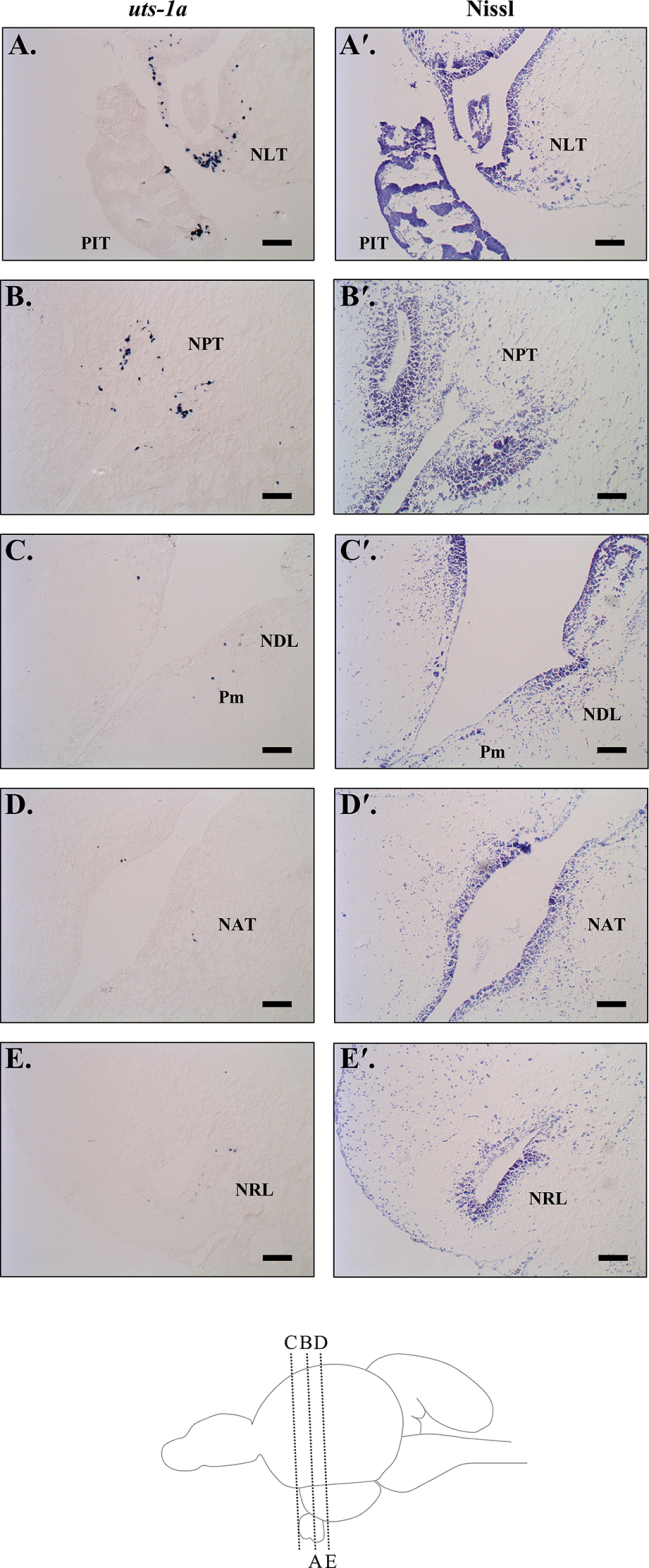
Urotensin 1a (*uts-1a*) mRNA localization in the diencephalon of Atlantic salmon (*Salmo salar*) as determined by *in situ* hybridization. **(A)** Symmetrical staining was observed in the lateral tuberal nucleus (NLT) on both sides of the infundibulum above the pituitary (PIT). **(B)** Positive staining was also observed above the infundibulum in the posterior tuberal nucleus (NPT). Scattered cells expressing *uts-1a* were observed along the third ventricle at the margin between the dorsolateral thalamic nuclei (NDL) and the magnocellular region of the preoptic nucleus (Pm; **C**), beside the infundibulum in the anterior tuberal nucleus (NAT; **D**), and in the nuclei beside the lateral recess (NRL; **E**). Adjacent sections that were Nissl stained with cresyl violet are included beside each *in situ* image (indicated with a ′) and a diagram of the brain showing the sagittal sectioning level for each image is included at the bottom of the figure. Scale bars = 100 µm.

Brain sections successfully hybridized the antisense DIG-labelled *uts-1a* riboprobe, whereas staining was absent from sections that were treated with the sense riboprobe (**Supplementary Figure 1**). We observed the strongest signal within the lateral and posterior tuberal nuclei (NLT & NPT; **Figures 9A, B**) in the hypothalamus. We also detected relatively weaker expression located along the border of the dorsolateral thalamic nuclei (NDL) and the magnocellular region of the preoptic nucleus (Pm; **Figure 9C**), as well as in the anterior tuberal and lateral recess nuclei (NAT & NRL; **Figures 9D, E**).

## 4 Discussion

**Figure 10:**
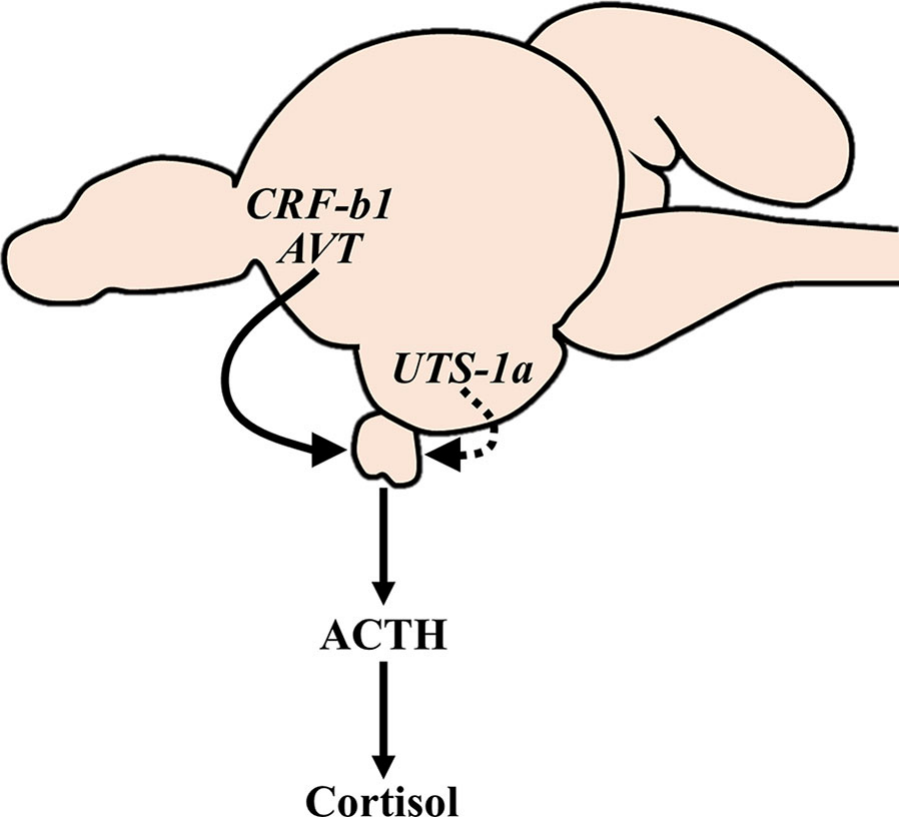
Proposed hypophysiotropic regulation of the HPI axis during smoltification of Atlantic salmon (*Salmo salar*). The HPI axis appears to be consistently stimulated (solid line) by AVT and CRF-b1 neurons in the preoptic area throughout late winter and spring, whereas UTS-1a neurons in the hypothalamus are progressively activated (dashed line) during smoltification. Note that the proposed mechanism is based on transcriptional changes that do not necessarily reflect changes in peptide synthesis and/or release. Additionally, the actions of these neuropeptides are diverse, and the observed changes are therefore unlikely to be limited to regulating cortisol levels. ACTH, adrenocorticotrophic hormone; AVT, arginine vasotocin; CRF-b1, corticotropin-releasing factor b1; HPI, hypothalamic-pituitary-interrenal; UTS-1a, urotensin 1a.

Cortisol coordinates many physiological changes that occur in advance of migration by diadromous fishes. However, the neuroendocrine regulation of cortisol levels during these periods has not been thoroughly evaluated. In the current study, we assessed the relationship between circulating cortisol and ACTH levels with transcriptional changes of the major neuroendocrine factors involved in the regulation of the HPI axis during smoltification in Atlantic salmon. Smolts displayed characteristic morphometric (reduced condition factor) and physiological (elevated branchial NKA activity and reduced physiological responses following transfer to seawater) changes consistent with preparation for seawater migration. Plasma cortisol levels and transcript abundance of *crf-b1* and *avt* in the preoptic area were all consistently higher in smolts compared to parr throughout the spring. However, when cortisol levels in smolts peaked in April, parallel changes indicative of an activated HPI axis were not observed. Instead, plasma ACTH levels and transcript abundance of several genes involved in regulating the HPI axis (e.g., *crf*-*r1a*, *pomc*-*b* in the pituitary; *uts-1a* in the hypothalamus) did not increase in smolts until May; after the peak in plasma cortisol levels. These combined data suggest that several hypophysiotropic factors are differentially involved in the regulation of plasma cortisol levels during smoltification (**Figure 10**) and subsequent seawater acclimation in Atlantic salmon.

In teleosts, activity of the HPI axis is thought to be primarily regulated by populations of CRF-b neurons located within the preoptic area of the brain (21, 22). Consistent with this role, smolts had higher cortisol levels and transcript abundance of preoptic *crf-b1* than parr across the spring/summer and following seawater exposure. Madaro et al. (84) also found that plasma cortisol levels and preoptic transcript abundance of *crf-b* (likely a combination of both *crf-b1* and *crf-b2*) were higher in post-smolt Atlantic salmon compared to parr. In addition, we found that preoptic transcript abundance of *avt*—another major regulator of the HPI axis in teleosts (21, 85, 86)—was upregulated in smolts and displayed very similar patterns to preoptic expression of *crf-b1*. As in mammals (87–89), AVT appears to work in combination with CRF in regulating ACTH release from the teleost pituitary (51, 90). However, AVT produced by preoptic neurons also contributes to circulating AVT levels (91) and circulating AVT can directly affect osmoregulation in peripheral tissues (92–95), making it difficult to parse out the direct versus indirect (e.g., HPI axis regulation) osmoregulatory effects of AVT. While we are not aware of any studies that have evaluated circulating AVT levels during smoltification in Atlantic salmon, seawater-acclimated rainbow trout have lower circulating AVT levels than trout held in freshwater (96–98). Consequently, it is likely that the elevated levels of preoptic *avt* observed in the current study reflect increased hypophysiotropic actions of AVT and not increased release from the pars nervosa. Overall, the similar seasonal patterns observed for *crf-b1* and *avt* expression strongly suggest that these peptides are regulated by the same external factor(s) during smoltification and indicate that both peptides may contribute to chronic increase in circulating cortisol levels observed during smoltification.

Despite parr displaying a robust increase in circulating ACTH and cortisol levels 24 h after transfer to seawater in the current study, similar transcriptional changes were not observed for preoptic expression of *crf-b1* and/or *avt*. Hyodo & Urano (98) found that transcript abundance of *avt* in the magnocellular neurons of the preoptic area was reduced in rainbow trout that had been transferred to seawater; likely reflecting a reduction in circulating AVT levels in seawater (96, 97). While we did not detect any significant changes in preoptic *avt* levels as Atlantic salmon acclimated to seawater in our study, transcript levels in both parr and smolts tended to be lower after 240 h of seawater acclimation compared to fish held in freshwater. These combined data suggest that AVT does not play a major role in regulating the HPI axis during seawater acclimation. In contrast, CRF-b has previously been implicated in the regulation of the HPI axis following seawater transfer in salmonids. Craig et al. (60) observed a marked increase in plasma cortisol levels and a ~2-fold increase in *crf-b1* transcript abundance in both the hypothalamus and preoptic area 24 h after transferring juvenile rainbow trout into seawater. This difference between our data and the findings of Craig et al. (60) may reflect potential species-specific and/or ontogenetic differences in the regulation of the HPI axis by the CRF system during seawater acclimation, but these possibilities need to be explicitly evaluated.

In contrast to the upregulation of preoptic *crf-b1* observed during smoltification, expression of *crf-a1* across the spring and summer tended to be higher in parr relative to smolts in both the hypothalamus and preoptic area. The teleost-specific peptide, CRF-a, was only discovered recently (36, 37) and current evidence for the involvement of CRF-a in the regulation of the HPI axis is mixed (39, 99–102). Of most relevance to the current study, Choi et al. (102) found that whole brain transcript abundance of *crf-a* (likely a combination of both *crf-a1* and *-a2*) increased in parallel with plasma cortisol, plasma ACTH, and pituitary expression of *pomc-a2* and -*b* when rainbow trout were transferred to seawater. Similarly, Lai et al. (39) reported that Atlantic salmon that had experienced 24 h of repeated exposure to either hypoxia, chasing, or the combination of hypoxia and chasing displayed parallel increases in circulating cortisol levels and transcript abundance of all CRF paralogs (*crf-a1*, *crf-a2*, *crf-b1*, and *crf-b2*) in the telencephalon (which likely included the majority of the preoptic nuclei) and *crf-a1* in the hypothalamus. In contrast, the patterns observed in the current study suggest that CRF-a is not involved in the regulation of the HPI axis during smoltification and seawater acclimation in Atlantic salmon because transcriptional changes in CRF-a were generally unrelated to—or in opposition of—changes in circulating ACTH and/or cortisol levels. Future work focusing on whether CRF-a expressing neurons project to the pituitary could help elucidate the potential regulatory involvement of CRF-a in the HPI axis.

We observed a strong divergence in hypothalamic *uts-1a* transcript abundance between parr and smolts during the spring smoltification period, with transcript levels increasing in smolts and decreasing in parr. Transcript levels of *uts-1a* in the hypothalamus also increased following seawater transfer, with immediate (<24 h) and delayed (96 h) upregulation in smolts and parr, respectively. Central distribution patterns of UTS-1 exhibit considerable variation among the teleost species evaluated to date. While UTS-1 is expressed in the ventral and lateral hypothalamus of zebrafish [*Danio rerio* (mRNA); (103, 104)], goldfish [protein; (44)] and white sucker [protein; (45)], UTS-1 expression is largely absent in the hypothalamus of medaka [*Oryzias latipes* (mRNA); (105)] and the African cichlid, *Astatotilapia burtoni* [mRNA; (106)]. Here, we found that expression of *uts-1a* in the diencephalon of Atlantic salmon was highest in the lateral and posterior tuberal nuclei (NLT and NPT), with additional staining observed near the margins of the magnocellular portion of the preoptic nucleus (Pm) and the dorsolateral thalamic nucleus (NDL), the anterior tuberal nucleus (NAT), and the lateral recess nucleus (NRL). These data support a role for UTS-1a in the regulation of the HPI axis in Atlantic salmon because neurons in these regions (especially the Pm and NLT) project to the pituitary in Atlantic salmon (23, 72) and UTS-1 neurons located in the NLT project to the rostral pars distalis in goldfish (44). The NLT is thought to play an important role in regulating corticotropes in teleosts because while lesions of either the preoptic area or the NLT cause reductions in circulating cortisol levels (46, 107), pituitary ACTH content is only reduced following lesioning of the NLT (107). These combined data support a potential role for hypothalamic UTS-1a in the regulation of ACTH synthesis in the pituitary. However, the CRF system is also involved in the regulation of thyrotropes and melanotropes in teleosts (21, 66, 108). Yulis et al. (45) only detected UTS-1 protein in the proximal pars distalis in white sucker (where thyrotropes are located), which is consistent with a role in regulating thyroid stimulating hormone. Overall, the physiological role(s) of UTS-1 and its relative contribution in regulating different pituitary cell-types in teleosts remains to be determined.

Consistent with McCormick et al. (8), we found that circulating ACTH levels in smolts were highest in May when the osmoregulatory capacity of smolts was greatest (e.g., gill NKA activity was highest). However, seasonal changes in circulating cortisol levels did not directly reflect differences in circulating ACTH levels. This disconnect is potentially a consequence of seasonal changes occurring within the interrenals. Cortisol synthesis is rate-limited both by levels of steroidogenic acute regulatory protein (StAR) which transports precursory cholesterol into the mitochondria of steroidogenic cells (31) and levels of cholesterol side-chain cleavage enzyme (P450scc/CYP11A) which catalyzes the initial conversion of cholesterol into pregnenolone (109). While it is likely that these proteins are upregulated during smoltification to facilitate enhanced cortisol synthesis, we are unaware of any study that has evaluated this possibility. However, previous work in coho salmon has shown that the sensitivity of the interrenals to ACTH increases in April (110), just prior to the peak of smolting. As such, greater amounts of cortisol are synthesized in the presence of equal ACTH concentrations during this period. This increase in ACTH sensitivity has been linked to elevated levels of both growth hormone (111) and thyroxine (112), which are also elevated during smoltification in Atlantic salmon (8, 9). Therefore, elevated circulating cortisol levels in smolts may be influenced by a combination of changes in circulating ACTH and altered interrenal sensitivity to ACTH. Additionally, despite ACTH being the primary regulator of cortisol synthesis (19, 22, 30), several other hormones can stimulate cortisol synthesis directly at the interrenals—although, usually to a lesser degree than ACTH (113, 114)—including UTS-1 (114–116). Unlike other vertebrates, fish also produce large amounts of CRF-b and UTS-1 in the caudal neurosecretory system (CNSS) located at the caudal tip of their spine, and these peptides are released from the urophysis directly into the caudal vein (117). Changes in salinity affect both the immunoreactive pattern of UTS-1 (118–121), as well as transcript abundance of CRF-b and UTS-1 in the CNSS (60, 122, 123). Additionally, expression of CRF-b and UTS-1 in the CNSS of European flounder (*Platichthys flesus*) changes over the course of the seasonal reproductive cycle, which corresponds with migrations between freshwater and seawater (124). Therefore, it is possible that CRF-related peptides produced by the CNSS are also contributing to the regulation of cortisol synthesis directly at the interrenals during smoltification.

Alternatively, the apparent disconnect between circulating cortisol levels and markers of HPI axis activity observed in the current study could also reflect seasonal changes in cortisol catabolism. Indeed, previous studies in coho salmon have found that plasma cortisol clearance rates display an inverse pattern to circulating cortisol levels (125, 126), with clearance rates increasing immediately following the seasonal peak in circulating cortisol levels. While our understanding of the regulatory mechanisms controlling rates of cortisol clearance/catabolism in teleosts is still developing (31, 127, 128), future work evaluating the regulation of major enzymes involved in cortisol catabolism (e.g., 11β-hydroxysteroid dehydrogenase type 2) could help elucidate the relative importance of cortisol synthesis versus clearance in determining plasma cortisol levels during smoltification.

Despite observing different patterns in circulating ACTH levels between parr and smolts across the spring and in response to seawater exposure, expression of several key components of the HPI axis within the pituitary (*crf-r1a, crf-r1b*, and *pc1*) exhibited similar changes in both parr and smolts. As such, the differences in circulating ACTH levels observed between parr and smolts could be a result of differences in post-transcriptional and/or post-translational regulation of genes in the corticotropes (and not due to changes in overall transcript abundance). For example, the activity of PC1 in mammals is dependent on autoproteolytic cleavage of its propeptide (129) and PC1 activity can be impaired by the presence of both its own propeptide (130) and the granin-like neuroendocrine secretory protein, proSAAS (131). While such mechanisms have not been evaluated in fish, the discrepancy between expression of genes involved in HPI axis regulation in the pituitary and circulating ACTH levels that we observed may be attributable to similar regulatory mechanisms.

Curiously, the only gene in the pituitary that differed between parr and smolts at the end of the spring was *pomc-b*. This is surprising because in rainbow trout *pomc-b* expression is restricted to melanotropes (132) and differences in *pomc-b* are therefore unlikely to be related to the regulation of the HPI axis. However, upregulation of *pomc-b* in the pituitary has previously been observed in smolting Atlantic salmon (8, 84) suggesting that POMC-derived peptides other than ACTH (i.e., melanocyte-stimulating hormones (MSH) and/or endorphins) are important during smoltification. For instance, desacetyl-α-MSH is a potent stimulator of lipolysis in salmonids (133, 134) and the observed upregulation of *pomc-b* may reflect an increase in desacetyl-α-MSH synthesis. Increased rates of lipolysis is one of the major metabolic changes that occurs during smoltification (135, 136) and the increase in *pomc-b* observed in the current study coincided with the substantial decline in condition factor of smolts (potentially indicative of increased lipolysis). Additionally, MSH stimulates the dispersion of pigments in melanophores (137) and the observed seasonal upregulation of *pomc-b* in smolts could also be associated with the development of the prominent dark margins on the caudal, dorsal and pectoral fins during smoltification (7). However, we are not aware of any evaluations of MSH levels in either the pituitary or circulation during smoltification and future work on melanotrope-derived peptides may clarify their role in smoltification.

In conclusion, we found that elevated cortisol levels during smoltification in Atlantic salmon likely reflect higher expression of *crf-b1* and *avt* in the preoptic area of the brain, as well as potential contributions by *uts-1a* originating from the NLT in the hypothalamus (**Figure 10**). Our data also suggest that an additional layer of complexity has been introduced into HPI axis regulation in salmonids because we observed several differences in the transcriptional regulation of salmonid-specific paralogs of CRF system components across the preoptic area, hypothalamus, and pituitary. Overall, our findings offer novel insights into the neuroendocrine factors that contribute to osmoregulation and highlight the complex, multifactorial regulation of the teleost HPI axis.

## Data Availability Statement

The original contributions presented in the study are included in the article/**Supplementary Material**. Further inquiries can be directed to the corresponding author.

## Ethics Statement

The animal study was reviewed and approved by USGS Eastern Ecological Science Center Institutional Animal Care and Use Committee (protocol LSC-9096).

## Author Contributions

All authors participated in the sampling of the fish and provided feedback on the manuscript. The study was designed by BC, SM, and NB. BC conducted the qPCR analysis, measured plasma ACTH levels, performed the *in situ* hybridization, analyzed the data, and wrote the first draft of the manuscript. AR performed the plasma cortisol assay. DH was responsible for most of the fish rearing and performed the gill NKA and osmolality assays. All authors contributed to the article and approved the submitted version.

## Funding

This work was supported by a Natural Sciences and Engineering Research Council of Canada (NSERC) Discovery grant provided to NB (RGPIN-2015-04498). BC was supported by a NSERC Doctoral Canadian Graduate Scholarship (CGS-D) and an Ontario Graduate Scholarship (OGS).

## Conflict of Interest

The authors declare that the research was conducted in the absence of any commercial or financial relationships that could be construed as a potential conflict of interest.

## Publisher’s Note

All claims expressed in this article are solely those of the authors and do not necessarily represent those of their affiliated organizations, or those of the publisher, the editors and the reviewers. Any product that may be evaluated in this article, or claim that may be made by its manufacturer, is not guaranteed or endorsed by the publisher.
