# Supplementary material for: Neuroendocrine Regulation of Plasma Cortisol Levels During Smoltification and Seawater Acclimation of Atlantic Salmon
Source: Front Endocrinol (Lausanne). 2022 Apr 21;13:859817. doi: 10.3389/fendo.2022.859817 (PMC9069684; doi:10.3389/fendo.2022.859817)
Supplement: Supplementary file 1 [file DataSheet_1.zip › Supplementary Material.docx]

Supplementary Material


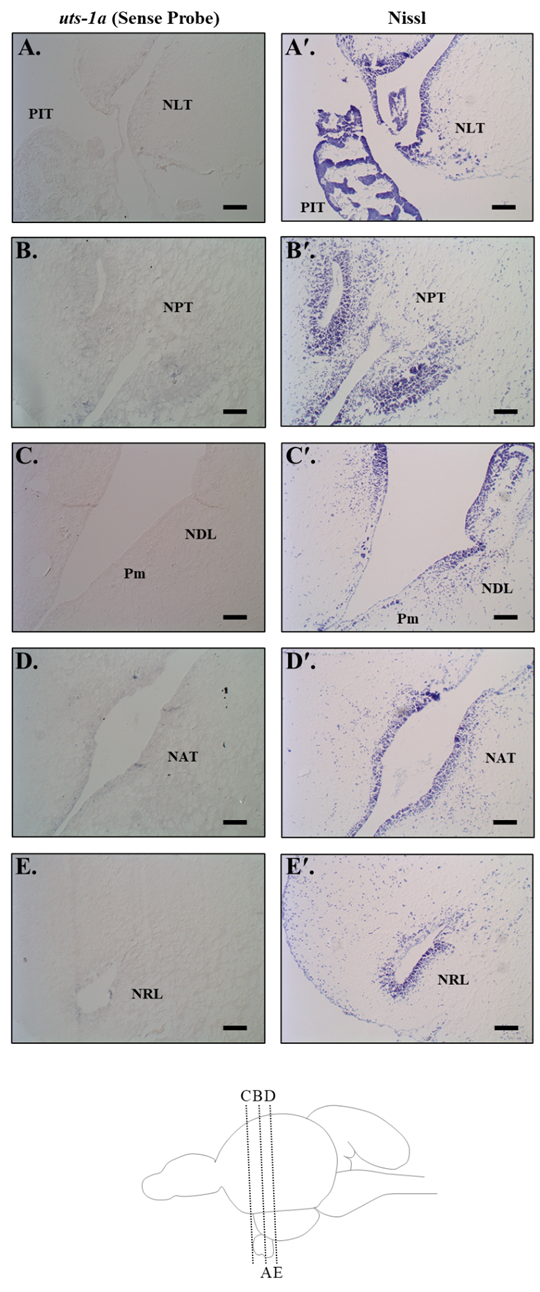


**Supplementary Figure 1.** Sections that were treated with the sense UTS-1a (uts-1a) riboprobe did not exhibit staining in either the (A) lateral tuberal nucleus (NLT), (B) posterior tuberal nucleus (NPT), (C) dorsolateral thalamic nuclei (NDL) and magnocellular region of the preoptic nucleus (Pm), (D) anterior tuberal nucleus (NAT), or (E) the nuclei beside the lateral recess (NRL). Adjacent sections that were Nissl stained with cresyl violet are included beside each in situ image (indicated with a ′) and a diagram of the brain showing the sagittal sectioning level for each image is included at the bottom of the figure. Scale bars = 100 µm.
